# Hemoperitoneum presenting with the use of a topical hemostatic agent in oocyte retrieval: a case report

**DOI:** 10.1186/1752-1947-6-395

**Published:** 2012-11-22

**Authors:** Amélie Chatrian, Clémentine Vidal, Véronique Equy, Pascale Hoffmann, Fabrice Sergent

**Affiliations:** 1 Service de Gynécologie-Obstétrique, CHU de Grenoble, Grenoble Cedex 09 38043, France

**Keywords:** Oocyte retrieval, hemoperitoneum, TachoSil^®^

## Abstract

**Introduction:**

Hemoperitoneum may occur from an ovarian puncture point after oocyte retrieval.

**Case presentation:**

We report a case of massive hemoperitoneum following transvaginal ultrasound-guided oocyte retrieval in a 33-year-old Caucasian woman. The bleeding required emergency laparoscopy because of active bleeding from the ovarian puncture point. Hemostasis was very difficult to achieve, and traditional operative procedures were not efficient. The only way to stop the bleeding was by using an absorbable fibrinogen and thrombin sealant sponge, which was applied around the ovary. During laparoscopy three pints of packed red blood were administered. No specific alteration of screening coagulation tests was found one month later.

**Conclusions:**

Hemostasis can be very difficult to achieve with traditional operative procedures. Topical hemostatic agents can be useful to preserve the ovary wherever possible.

## Introduction

We report a case of massive hemoperitoneum requiring emergency laparoscopy because of active bleeding from the ovarian puncture point.

## Case presentation

A 33-year-old Caucasian woman was referred to our facility for primary infertility resulting from her partner, who had secretory azoospermia. Our patient had regular menstrual cycles. Her body mass index was estimated to be 21. Previous hormonal and ultrasonographic evaluations confirmed ovulatory cycles. Obstructed tubes were seen on X-ray hysterosalpingography. During the hysterosalpingography, there was no free spillage of contrast material into the peritoneal cavity. Laparoscopy showed tubal patency. Our patient did not have any post-surgical complications. Six intracervical inseminations with donor sperm failed. Four intrauterine inseminations with donor sperm also failed. Finally an *in vitro* fertilization with donor sperm was performed. A first transvaginal oocyte puncture had been performed eight months previously without any complications. Induction of ovulation was initiated by using pure follicle-stimulating hormone administrated intramuscularly.

When more than three follicles ≥17mm in diameter were present, 5000IU human gonadotropin was administrated intramuscularly. Our patient had not taken any other medication before the oocyte puncture. Transvaginal ultrasonography-guided oocyte retrieval was performed 35 hours after the human chorionic gonadotropin injection had been given. The follicles were retrieved by sequential puncture without reinsertion of the needle through the vaginal wall, so only one puncture was made on each side. A total of 13 oocytes were retrieved without using solutions for flushing. At the end of the procedure there were no ultrasonographic signs of bleeding and our patient was kept in the department for observation.

Three hours after the procedure our patient stated she had abdominal pain. Her blood pressure was normal, and her pulse rate was 70 beats per minute. There was no fever. An abdomen rebound defense appeared. Her hemoglobin level was 99g/L and hematocrit was 29 percent. A transvaginal sonography revealed a blood collection in the pouch of Douglas and in the Morisson space.

**Figure 1:**
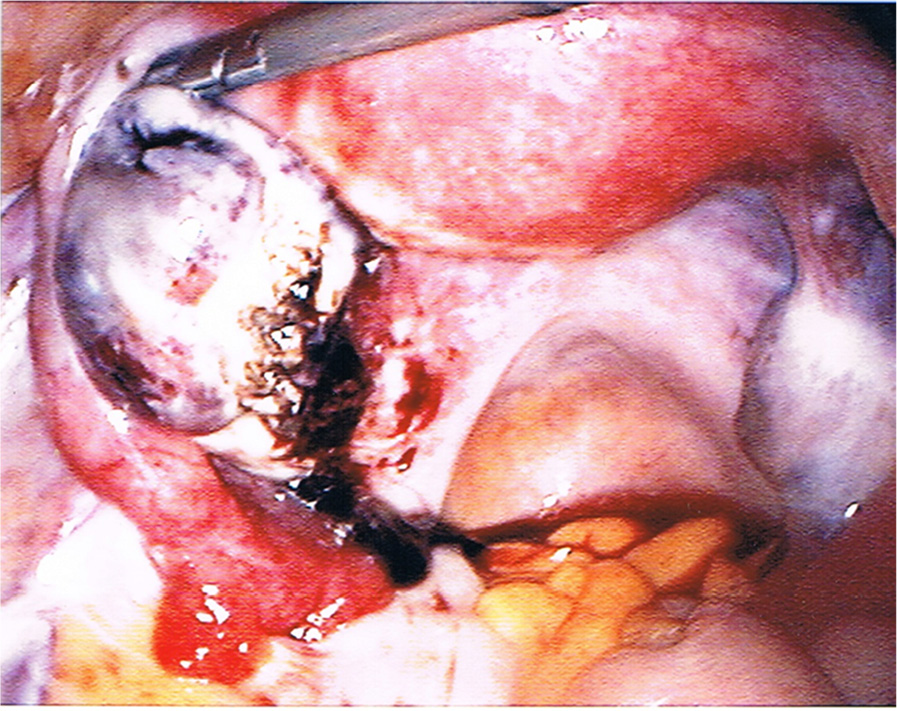
Active bleeding from the left ovarian puncture point.

**Figure 2:**
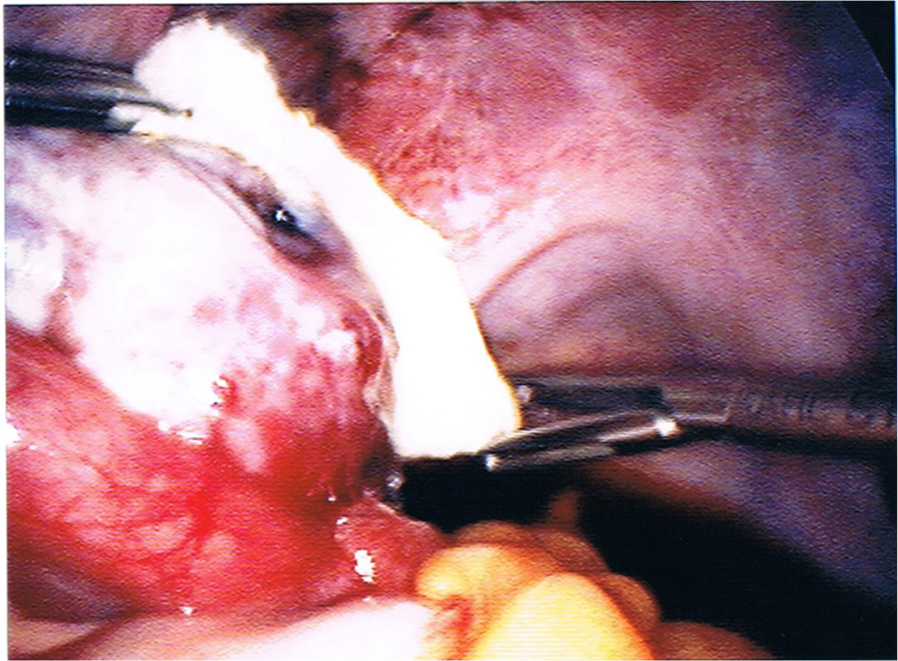
Use of fibrinogen and thrombin sealant sponge.

Six hours after the procedure, her pain increased and our patient became pale. Her blood pressure and pulse rate were normal. The abdomen rebound defense increased. Her hemoglobin level was 90g/L and hematocrit was 26 percent. Coagulation tests showed normal prothrombin and partial prothrombin times. Transvaginal and transabdominal sonography showed massive hemoperitoneum. This severe bleeding required emergency laparoscopy, seven hours after the oocyte retrieval, finding a massive hemoperitoneum of 2.5L. At the beginning of the procedure her hemoglobin was 77g/L with the HemoCue^®^ system (the diagnosis of anemia can be accomplished by assessing hemoglobin concentration with point-of-care testing devices as the HemoCue^®^ test system, HemoCue France, 77100 MEAUX). No pelvic vessel lesions had been observed. Hemoperitoneum was evacuated and active bleeding from the left ovarian puncture point was noted (Figure 1). Hemostasis was difficult to achieve. Traditional operative procedures such as bipolar coagulation, use of hot water or use of oxidized cellulose hemostats (Surgicel^®^) were not efficient. Eventually the only way to stop bleeding was human fibrinogen and thrombin sponge (TachoSil^®^, Nycomed, Denmark), which was applied around the ovary (Figure 2). During laparoscopy three pints of packed red blood were administrated.

Her post-operative hemoglobin level was 111g/L, and hematocrit was 32 percent.

Our patient was discharged from hospital after two days. A transvaginal sonography procedure was performed within 10 days, and this did not show any blood collection. No specific alteration of screening coagulation tests was found one month later.

All embryos were allocated to a cryopreservation protocol and at this time our patient has not had the embryos transferred.

## Discussion

Ultrasonographically-guided vaginal oocyte recovery, used to collect oocytes for in vitro fertilization, is a relatively atraumatic method with rare complications. It was first described by Wikland *et al*. in 1985 [1]. This procedure is fast and easy, and has proved to be efficient with minimal discomfort for patients. It can be performed with local anesthesia. However, the aspiration needle may injure the fine vascular network of blood vessels on the ovarian surface and theca interna layer or damage the adjacent pelvic organs. The subsequent complications are vaginal hemorrhage (8.6 percent), vaginal hemorrhage with loss of more than 100ml of blood (0.8 percent), hemoperitoneum (0.07 percent), punctured iliac vessels (0.04 percent), and post-operative pelvic infection (0.6 percent) [2].

Severe intraperitoneal bleeding was defined as a decrease in hemoglobin level, hematocrit level and blood pressure, and a medium to large volume of pelvic fluid.

Few cases of acute hemoperitoneum have been reported in the literature. To reduce their occurrence and severity, the patient must be kept in the ward for at least four hours post-procedure to control blood pressure, pulse rate, vaginal bleeding, diffuse abdominal pain or vomiting. Overall, most patients tolerate the pain. The pain level increases with the number of oocytes retrieved [3].

Blood loss that should be considered ‘normal’ in the first 24 hours after non-complicated transvaginal oocyte retrieval has been estimated to be approximately 230ml.

The reduction of hemoglobin has been estimated to be 1.6±0.8g/100ml. Several parameters were considered: the number of follicles aspirated, the number of oocytes collected, the pre-ovulatory E2 levels, and the duration of the procedure; this was without correlation with the amount of blood loss [4].

Hemoperitoneum may occur from the ovarian puncture point or from direct damage to pelvic blood vessels or pelvic organs. It may also occur as a result of bleeding of small intra-follicle vessels during the flushing of the follicular bed with solutions containing heparin [5]. In our patient, no heparin-containing solutions were used for flushing.

Coagulation occurs when a damaged blood vessel interacts with clotting proteins and platelets to form a stable platelet-fibrin plug. Abnormalities of any of these factors may result in clinically significant bleeding. Battaglia *et al*. [6] report a massive hemoperitoneum in a case of coagulation factor XI deficiency and EI-Shawarby *et al*. [7] in a case of thrombocythemia. Leaner patients may be at a much higher risk for acute hemorrhage [8,9]. Pregnancy outcome seems not to be affected by intraperitoneal bleeding and subsequent surgery [8].

TachoSil^®^ is a collagen sponge coated with human coagulation factor fibrinogen and thrombin. It is indicated as an adjunct to hemostasis when control of bleeding by standard surgical techniques (such as suture, ligature or cautery) is ineffective or impractical, to promote tissue sealing and for suture support in vascular surgery.

In the literature, a partial resection of ovary or a salpingoophorectomy was often performed because of oozing from the surface of the ovary [6,7].

Hemostasis can be very difficult to achieve with traditional operative procedures, and the ovary must be preserved whenever possible. Then a topical hemostatic agent can be utilised to avoid oophorectomy.

Finally, in our patient’s case, her ovary was preserved by using an absorbable fibrinogen and thrombin sealant sponge.

## Conclusions

Severe abdominal pain after oocyte retrieval should be taken seriously. Severe hemorrhagic complications may rarely occur. Therefore, the most important factor to prevent intraperitoneal bleeding is to avoid repeat penetrations of the ovary and follicle. When active bleeding from an ovary is observed, hemostasis is often difficult to achieve. Use of human fibrinogen and thrombin sponges may help avoid oophorectomy.

## Consent

Written informed consent was obtained from the patient for publication of this case report and any accompanying images. A copy of the written consent is available for review by the Editor-in-Chief of this journal.

## Competing interests

The authors declare that they have no competing interests.

## Authors’ contributions

All authors read and approved the final manuscript. AC, CV and FS analyzed and interpreted the patient data. VE and PH were contributors to the writing of the manuscript.
